# Unraveling the role of mitochondrial dysfunction in diabetic kidney disease: insights and interventions

**DOI:** 10.3389/fphar.2025.1618418

**Published:** 2026-01-06

**Authors:** Zhenliang Fan, Puchang Luo, Yuancheng Gao, Hongzhen Ma, Junfen Fan, Shriya Sanan, Peipei Zhang, Keda Lu, Hong Xia

**Affiliations:** 1 Department of Nephrology, the First Affiliated Hospital of Zhejiang Chinese Medical University (Zhejiang Provincial Hospital of Chinese Medicine), Hangzhou, Zhejiang, China; 2 Zhejiang Key Laboratory of Research and Translation for Kidney Deficiency-Stasis-Turbidity Disease, Hangzhou, Zhejiang, China; 3 Zhejiang-Macau International Joint Laboratory of Integrated Traditional Chinese and Western Medicine for Nephrology and Immunology, Hangzhou, Zhejiang, China; 4 Department of Nephrology, Linping Hospital of Integrated Traditional Chinese and Western Medicine, Hangzhou, China; 5 Zhejiang Chinese Medical University, Hangzhou, China; 6 Department of Nephrology, the Third Affiliated Hospital of Zhejiang Chinese Medical University, Hangzhou, China

**Keywords:** diabetic kidney disease, mitochondrial dysfunction, oxidative stress, therapeuticinterventions, mitochondrial biogenesis, clinical translation

## Abstract

The incidence of Diabetic Kidney Disease (DKD) is rising globally, paralleling the increasing prevalence of diabetes mellitus (DM). As DM spreads worldwide, DKD becomes a significant and growing complication, challenging healthcare systems. DKD is a leading cause of end-stage renal disease (ESRD), requiring costly renal replacement therapies. Mitochondria are vital for cellular energy production *via* oxidative phosphorylation (OXPHOS), playing a pivotal role in DKD pathogenesis through dysfunction in energy metabolism, reactive oxygen species (ROS) generation, and mitochondrial dynamics. Emerging evidence highlights the crucial role of mitochondrial dysfunction in the pathogenesis and progression of DKD. This review elucidates the intricate relationship between mitochondrial dysfunction and DKD pathophysiology, emphasizing mechanisms such as impaired OXPHOS, excessive ROS production, and disrupted mitochondrial biogenesis. We critically analyze therapeutic interventions, including preclinical compounds, repurposed clinical drugs, and experimental molecules, highlighting their efficacy, limitations, and clinical translation challenges. Emerging evidence suggests novel mitochondrial-targeted therapies may mitigate DKD progression, though controversies, such as inconsistent PGC-1α expression, warrant further investigation. By integrating molecular insights with clinical perspectives, this review aims to guide future research and therapeutic development for DKD.

## Introduction

The incidence of DKD is on a steady and concerning rise globally, mirroring the escalating prevalence of diabetes itself. As DM continues its relentless advance across populations worldwide, DKD emerges as a significant and increasingly prevalent complication, presenting a formidable challenge to healthcare systems worldwide. DKD develops in approximately 40% of patients who are diabetic and is the leading cause of CKD worldwide (Alicic et al., 2017). DKD is linked to considerable morbidity and mortality, accounting for 34% of all CKD deaths among men and 36% among women (Ghose et al., 2024). Functioning as the cellular battery unit, mitochondria synthesize adenosine triphosphate (ATP) from ADP *via* OXPHOS, accounting for more than 90% of the energy production in the human body. OXPHOS, also known as an electron transport-linked metabolic pathway, is dependent on the oxidative reactions that utilize the energy of nutrients to generate ATP through electron transport chain (ETC.) The process needs the help of electron carriers like NADH or FADH_2_, complex I, II, III, IV, coenzyme Q (CoQ), and cytochrome C (Cyt C).

Mitochondria plays a pivotal role in the control of cellular redox and energy homeostasis, and therefore a major source of intracellular oxidative stress (San-Millán, 2023; Ratliff et al., 2016; Forbes and Thorburn, 2018). Oxidative stress can be classified into two major categories: reactive oxygen species (ROS) and reactive nitrogen species (RNS). ROS are intrinsic to cellular function and are consistently present at low levels in healthy cells. Oxidative stress arises from both unregulated ROS production and inadequate ROS removal by antioxidant systems (Sakashita et al., 2021).

The kidney, a highly metabolic organ, relies on mitochondria for energy-intensive processes such as solute reabsorption, waste elimination, and electrolyte balance, primarily through ATP production *via* OXPHOS (Bhargava and Schnellmann, 2017). Hence, it is suggested that mitochondrial dysfunction significantly contributes to the development and advancement of kidney disorders, such as DKD (Mise et al., 2020; Wei and Szeto, 2019; Ito et al., 2022).

This review synthesizes the dynamic interplay between mitochondrial dysfunction and DKD, critically evaluating therapeutic strategies and addressing translational challenges to inform clinical practice and future research.

## Injury factors to mitochondrial function

### Overview of mitochondrial roles

The prominent physiological role of mitochondria is to produce ATP *via* OXPHOS. Furthermore, mitochondria are involved in bio-processes such as mitochondrial ROS generation, biogenesis, fission, fusion and autophagy (Yang et al., 2024). Any impairment in these processes can lead to mitochondrial dysfunction.

### Key mechanisms of mitochondrial injury

**FIGURE 1:**
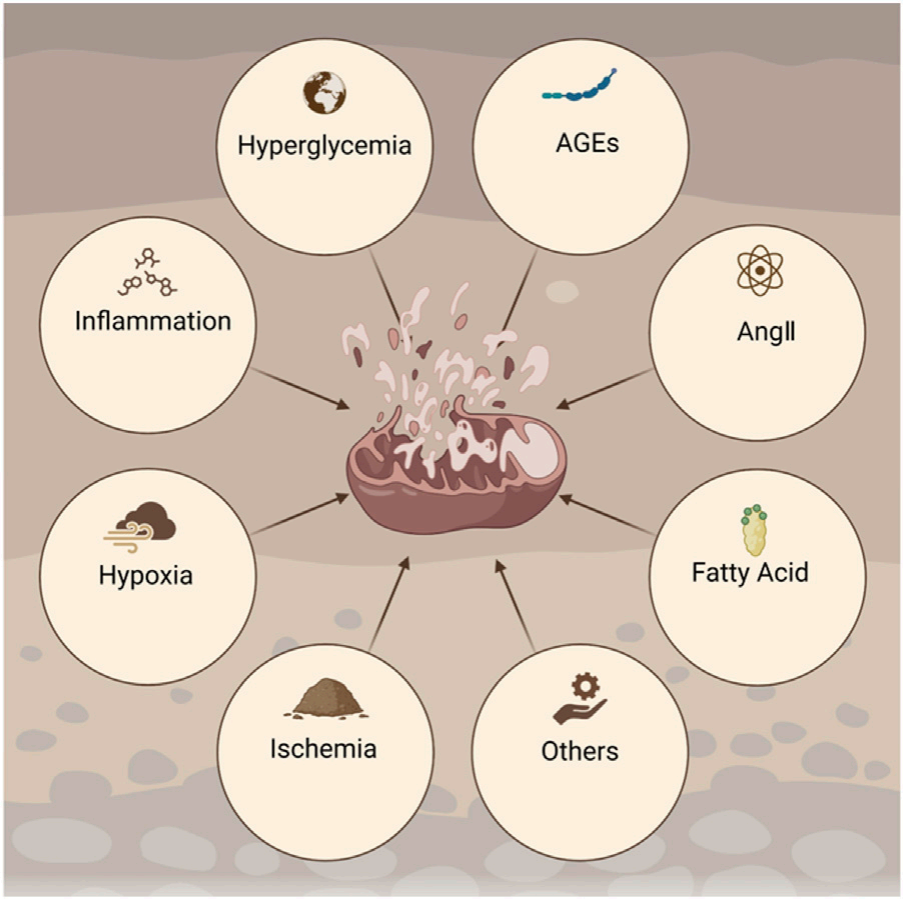
Factors involved in the pathogenesis of DKD. AGEs, advanced glycation end products; AngII, angiotensin II. Created with BioRender.com.

Mitochondrial dysfunction in DKD arises from multiple stressors, including hyperglycemia-induced oxidative stress, altered mitochondrial dynamics, and impaired mitophagy. These mechanisms are summarized below to clarify their roles in renal injury (Figure 1).

## Mitochondrial dysfunction involved in diabetic nephropathy

### Excessive mitochondrial oxidative stress

#### Altered oxidative phosphorylation

Indicators of OXPHOS function and efficiency encompass oxygen consumption rate (OCR), ATP generation, membrane potential, and the assessment of individual complexes (their activity and formation) (Ito et al., 2022). Typically, it has been noted that OCR in the renal cortex rises during early stages of DKD, subsequently declining as the disease advances. Conversely, OCR in glomeruli and podocytes decreases during both early and late phases of the disease (Mise et al., 2020; Ito et al., 2022). The reduced activation of OXPHOS appears to play a role in DKD, as suggested by findings showing associations between certain genetic mutations in OXPHOS, such as single-nucleotide polymorphisms (SNPs) in coenzyme Q5 (COQ5) and cytochrome c oxidase subunit 6A1 (COX6A1), and DKD in humans (Swan et al., 2015).

#### Excessive production of ROS

In kidney cells, elevated glucose levels result in heightened activity of protein kinase C (PKC), prompting the production of endothelial nitric oxide synthase (eNOS) and enhancing the NO during the early phases of DKD (Geraldes and King, 2010). Elevated levels of NO stimulates vascular endothelial growth factor (VEGF), ultimately causing impairment in endothelial function (Kanwar et al., 2011). Endothelial cell dysfunction inhibits mitochondrial function and boosts ROS production. ROS are generated as part of the normal functioning of the mitochondrial ETC (mtETC) (Huang et al., 2020). However, when the mtETC malfunctions, there is an excessive production of ROS, leading to oxidative stress and cellular damage in the kidney (Ito et al., 2022).

#### Various mechanisms: excessive mitochondrial oxidative stress contributing to kidney damage

Mitochondrial oxidative stress contributes to kidney damage through various mechanisms, (Huang et al., 2020), including: (1) Elevated glucose levels activate the c-Jun N-terminal kinase (JNK)- calcium/calmodulin-dependent protein kinase II (CaMKII)- fission factor 1 (FIS1) pathway, leading to mitochondrial fragmentation, heightened production of ROS, and increased activation of JNK. This heightened JNK activity exacerbates podocyte apoptosis and damages renal tubular cells in mice (Zhang Y. et al., 2018) (2) Mitochondrial dysfunction hampers the adenosine monophosphate-activated protein kinase (AMPK)- Sirtuin-1 (SIRT1) - peroxisome proliferator-activated receptor-gamma coactivator-1α (PGC-1α) pathway, leading to a decrease in podocyte autophagy. As a result, podocyte damage accumulates, leading to elevated levels of albuminuria (Akhtar and Siragy, 2019) (3) Overproduction of ROS triggers the generation of transforming growth factor-beta (TGF-β), suppresses nitric oxide (NO) levels, and leads to renal fibrosis and damage to endothelial cells (Honda et al., 2019) (4) Excessive ROS hinder mechanistic target of rapamycin complex 1 (mTORC1) and AMPK function, affecting PGC-1α activation. PGC-1α, vital for mitochondrial biogenesis and ROS repair, declines, reducing mtDNA. This leads to decreased mtETC protein synthesis, ROS buildup, and ongoing mtDNA damage, causing kidney impairment (Amorim et al., 2019).

#### Various mechanisms: excessive mitochondrial oxidative stress contributing to DKD

In DKD, oxidative stress response intertwines with other stress reactions like advanced glycation end-products (AGE) formation and hypoxia. Prolonged hyperglycemia triggers AGE production, inducing oxidative stress by binding to AGE receptors. Mitochondria are known to be susceptible to oxidative damage, and increased levels of ROS, as well as the presence of AGEs, can impair mitochondrial function. This dysfunction can further exacerbate oxidative stress, creating a feedback loop that contributes to the progression of conditions like DKD. Mitochondrial oxidative stress exacerbates the DKD cycle (Ito et al., 2022): (1) High glucose boosts ATP demand, increasing ROS production by mtETC; (2) ROS overproduction damages biomolecules; (3) ROS inhibits mitochondrial growth by downregulating PGC-1α, reducing mtDNA; (4) Diminished mtDNA synthesis impairs electron transport, escalating superoxide generation, further harming mtDNA; (Santos et al., 2011); (5) ROS and impaired mtDNA accumulate over time, resulting in damage to podocytes and renal tubular cells, further worsening renal fibrosis (Huang et al., 2020).

#### Indirect mechanisms of oxidative stress-induced injury in DKD

**FIGURE 2:**
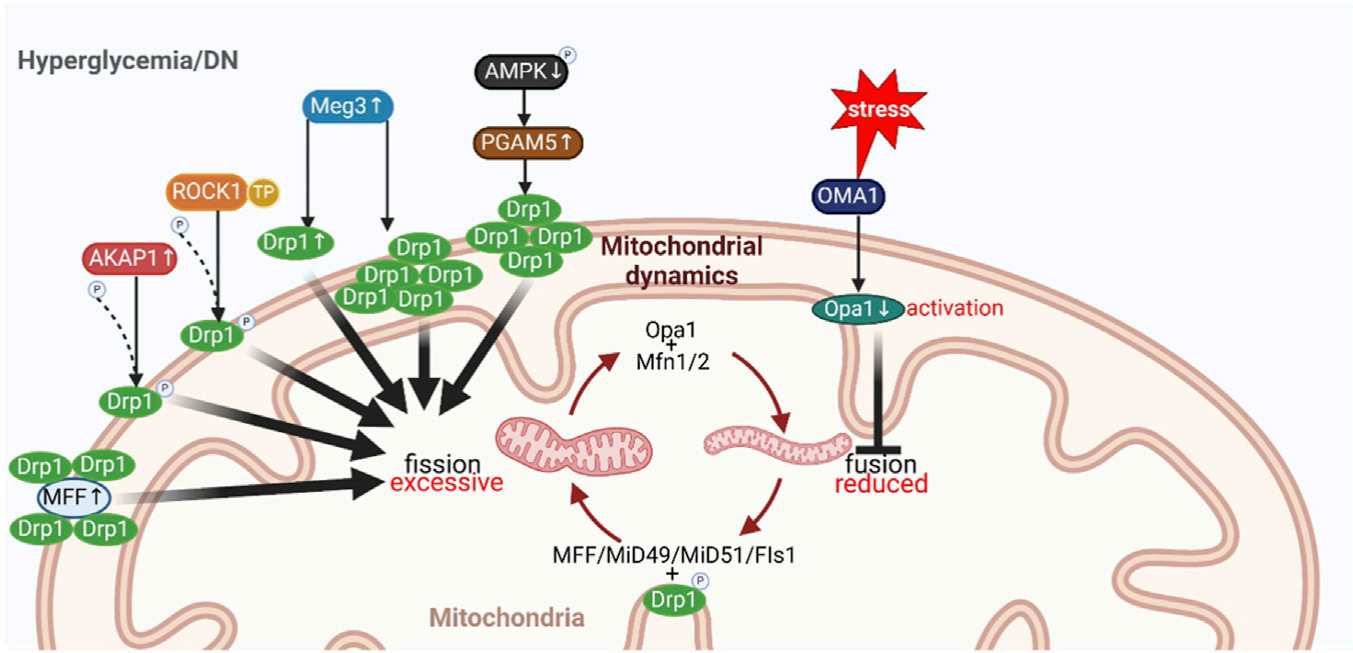
Key mediators of resident renal cells injury in diabetes nephropathy. The resident renal cells include the podocytes, mesangial cells, endothelial cells and renal tubular epithelial cells. As shown, several possible mechanisms lead to kidney injury implicated in DKD. ROS, reactive oxygen species; Cyt c, Cytochrome c; Apaf-1, apoptotic protease activating factor-1; NF-κB, nuclear factor-kappaB; MCP-1, monocyte chemoattractant protein-1; PKC, protein kinase C; VEGF, vascular endothelial growth factor; Rac1, Ras-related C3 botulinum toxin substrate 1; ERK, extracellular signal-related kinases; eNOS, endothelial nitric oxide synthase; NO, nitric oxide; ADAM17, ADAM metallopeptidase domain 17; ECM, extracellular matrix; PKB, protein kinase B; TRPC6, transient receptor potential cation channel, subfamily C, member 6; EMT, epithelial-mesenchymal transition; ICAM-1, intercellular adhesion molecule 1; TGF- β, transforming growth factor- β; IL-1 β, interleukin-1 β; IL-6, interleukin-6; MAPK, mitogen-activated protein kinase; CTGF, connective tissue growth factor. Created with BioRender.com.

Oxidative stress can also induce kidney damage indirectly. For instance, ROS can increase in number by the overexpression of tumor necrosis factor (TNF), but overloaded ROS can drive nuclear factor kappa-B (NF-κ B) downstream signal transduction, which is closely referred to many complications of diabetes (Jha et al., 2016; Jin et al., 2023). Besides, diversified signaling pathways, such as AGE-receptor for advanced glycation end products (RAGE), (Nabi et al., 2023), Kelch-like ECH-associated protein 1(Keap1)- nuclear respiratory factor 2 (NRF2), (Zhu et al., 2021), AMPK/SIRT1, (Gao and Wu, 2022), SIRT1-forkhead transcription factor O (FOXO), (Jalgaonkar et al., 2022), SIRT, (Qiu et al., 2022), and high mobility group box-1 protein (HMGB1) (Jin et al., 2023) could also be induced by oxidative stress (Figure 2). It has been shown that PGC-1α is the key modulator of ROS pathway in the pathogenesis of DKD (Guo et al., 2015; Wu et al., 2015; Al-Kafaji et al., 2016). PGC-1α ameliorates kidney fibrosis in DKD mice by mitigating oxidative stress in podocyte and mesangial cells (Zhang L. et al., 2018).

### Impaired mitochondrial biogenesis

#### Altered mitochondrial biogenesis in DKD

Mitochondrial biogenesis is the physiological response to external stimuli to maintain mitochondrial homeostasis (Chen et al., 2022). Mitochondrial biogenesis and dynamics are associated with renal mitochondrial dysfunction and the pathophysiological development of DKD (Song et al., 2022). Cells manage rising energy requirements by boosting mitochondrial biogenesis, a process wherein functional mitochondria are produced through the duplication of mitochondrial DNA (mtDNA) followed by binary fission (Ito et al., 2022). In DKD patients, the enhancement of mitochondrial biogenesis may be a protective to meet the high energy demand in kidney. However, some contrasting studies suggest that mitochondrial biogenesis decreases in diabetic mice (Bugger et al., 2009; Dugan et al., 2013; Coughlan et al., 2016a). A study of the development of rat model in DKD suggested that mitochondrial biogenesis is a common early compensatory event that occurs in conjunction with kidney hyperfiltration and a decline in mitochondrial ATP content but declines following the progression of DKD (Coughlan et al., 2016b).

#### A pivotal factor - PGC-1α, in the network of mitochondrial biogenesis

**FIGURE 3:**
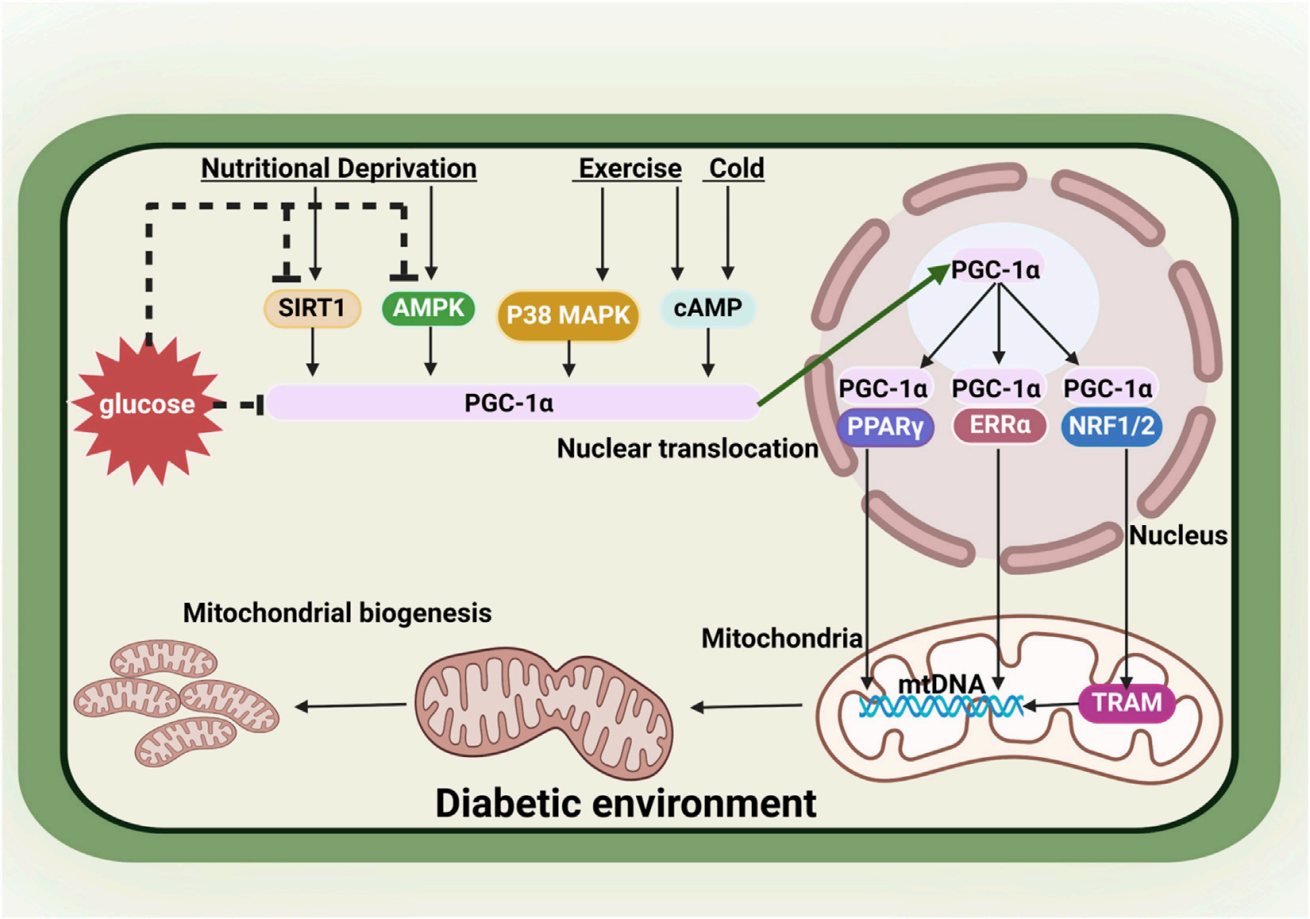
The regulation of mitochondrial biogenesis and changes affected by diabetic environment. PGC-1α serves as a key regulator of mitochondrial biogenesis. Diabetic environment inhibits PGC-1α by suppressing PGC-1α activators AMPK and SIRT, or suppressing PGC-1α directly. SIRT1, sirtuin 1; AMPK, adenosine monophosphate-activated protein kinase; MAPK, mitogen-activated protein kinase; cAMP, cyclic adenosine monophosphate; PGC-1α, peroxisome proliferator-activated receptor γ coactivator1α; PPARγ, peroxisome proliferator-activated receptor γ; ERRα, estrogen-related receptor α; NRF1/2, nuclear respiratory factor 1/2; TFAM, mitochondrial transcription factor A; mtDNA, mitochondrial DNA. Created with BioRender.com.

Mitochondrial biogenesis is regulated by a variety of factors, much like a network. This multifaceted process comprises a network of therapeutic targets, including upstream sensors and downstream effectors. PGC-1α serves as a pivotal regulator in this intricate network, integrating upstream signals to launch a downstream mitochondrial gene programs facilitating mitochondrial biogenesis (Fernandez-Marcos and Auwerx, 2011). The upstream sensors can activate PGC-1α, including AMPK, SIRT1, calcium/calmodulin-dependent protein kinase IV (CaMKIV), p38 mitogen-activated protein kinase (MAPK), and NO. PGC-1α then regulates mitochondrial biogenesis by modulating various downstream transcription factors, including mitochondrial transcription factor A (TFAM), nuclear respiratory factor 1 (NRF1), NRF2, peroxisome proliferator-activated receptor gamma (PPARγ), estrogen-related receptor α (ERRα) (Ventura-Clapier et al., 2008; Scarpulla, 2011). The transcriptional regulation and post-translational modifications of PGC-1α introduce substantial challenges in considering PGC-1α as a direct target for pharmacological interventions. The network of mitochondrial biogenesis undergoes corresponding changes in a diabetic environment (Figure 3) (Clark and Parikh, 2021)

#### Altered PGC-1α in DKD

In DKD, there’s a notable decrease in the expression of PGC-1α within the intrinsic renal cells, triggering a series of harmful consequences. This encompasses diminished mitochondrial biogenesis, increased levels of oxidative stress in mitochondria, disrupted mitochondrial dynamics, and irregular mitophagy. This leads to structural and functional irregularities in the mitochondrial network. Conversely, maintaining high levels of PGC-1α expression proves promising in preserving mitochondrial balance within renal cells, showing significant potential for kidney protection in animal models with DKD (Ye et al., 2024). However, a study by Li et al. showed a heightened PGC1-α expression in the context of DKD, which was concomitant with the occurrence of proteinuria and the spontaneous development of renal pathological alterations (Li et al., 2017). This study leads a noteworthy revelation that the therapeutic efficacy of targeting podocyte PGC1-α in the context of DKD may depend on a specific temporal window. There is the necessity for additional experiments to find out both the efficacy and safety of interventions targeting podocyte PGC1-α level in the context of DKD.

### Mitochondrial dynamics abnormalities

#### What is mitochondrial dynamic?

Mitochondrial dynamics encompasses the processes of fission, fusion, mitophagy, and transport, all of which are essential for maintaining optimal function in signal transduction and metabolism (Chen et al., 2023). Mitochondria are highly motile organelles that undergo carefully regulated processes of division and fusion. Mitochondrial fission and fusion events enable energy demands to be met and provide mitochondrial quality control; disruption of these events in diabetes prevents elimination of damaged mitochondria and exacerbates ATP deficits (Forbes and Thorburn, 2018). Mitochondrial fusion is orchestrated by the long isoforms of optic atrophy protein 1 (OPA1), which primarily mediate inner mitochondrial membrane fusion, and the mitofusins (MFN1 and MFN2), responsible for outer mitochondrial membrane fusion (Mise et al., 2020; Ito et al., 2022). Conversely, mitochondrial division, known as fission, is facilitated by dynamin-1-like protein (DRP1) and its associated receptors such as FIS1, mitochondrial fission factor (MFF), and mitochondrial dynamics proteins of 49 and 51 kDa (MID49 and MID51). In the context of DKD, mitochondria, as dynamic components within cells, continually undergo both fission and fusion processes to adapt to changes in their environment. Fission is crucial for the creation of new mitochondria and the isolation of damaged ones, while fusion leads to the formation of elongated or tubular mitochondria, enabling the exchange of materials between them and potentially compensating for any functional impairments (Suárez-Rivero et al., 2017).

#### Excessive fission and reduced fusion in DKD

**FIGURE 4:**
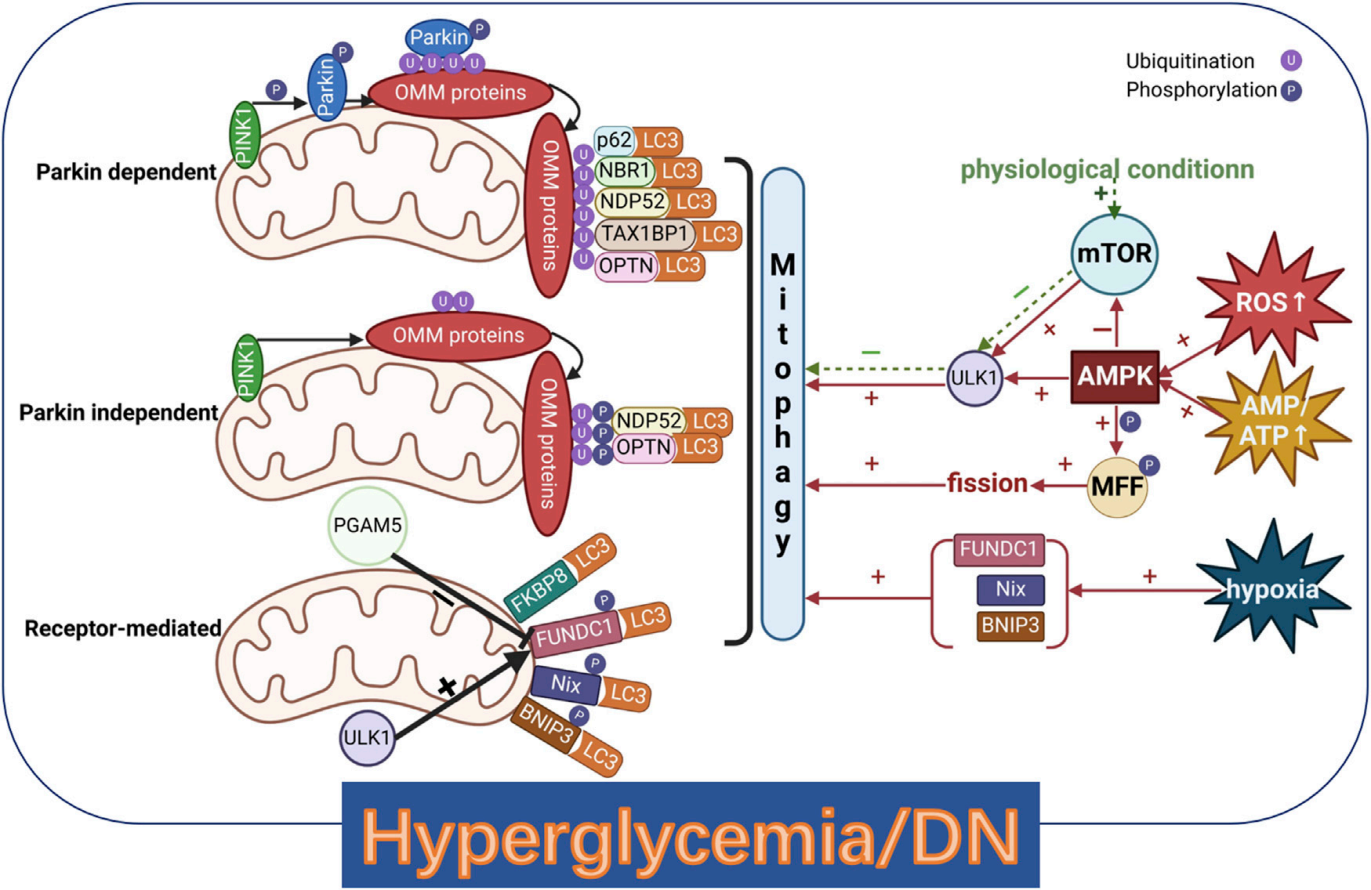
Under hyperglycemia or diabetic disease environment, renal mitochondrial dynamics disorder. Drp1, dynamin-related protein 1; MFF, mitochondrial fission factor; ROCK1, Rho-associated kinase 1; TP, thromboxane receptor; AKAP1, a kinase-anchored protein 1; Meg3, maternally expressed gene 3; AMPK, adenosine monophosphate-activated protein kinase; PGAM5, phosphoglycerate mutase family member 5; Opa1, optic atrophy protein 1; Mfn1/2, mitochondrial fusion protein 1/2; FIs1, mitochondrial fission 1. Created with BioRender.com.

Imbalanced mitochondrial dynamics, characterized by excessive fission and reduced fusion, contribute to mitochondrial fragmentation in DKD, thus contributing to disease progression (Figure 4) (Yang et al., 2024) Fragmented mitochondria are more prone to dysfunction and apoptosis, further contributing to renal damage (Forbes and Thorburn, 2018). Even though there was an observed rise in mitochondrial fission and fusion factors like the long isoforms of OPA1, MFN1, MFN2, and MFF during the initial stages of DKD, mitochondria remained fragmented consistently from the early to late stages in rats injected with STZ (Ma et al., 2019). Human kidney biopsies from patients with DKD also revealed fragmented mitochondria in both podocytes and proximal tubular cells (Jiang et al., 2019; Ma et al., 2019). Consistent with increased fission and decreased fusion, DRP1 and FIS1 expression was increased, while MFN2 expression was shown to be decreased in tubules in the latter study (Ito et al., 2022). A-kinase anchoring protein (AKAP1) plays a pivotal role in the pathogenesis and progression of hyperglycemia (HG) -induced podocyte injury by disrupting mitochondrial dynamic homeostasis through the regulation of DRP1 phosphorylation in human podocytes (Chen Z. et al., 2020). Some findings indicate that upregulated expression of the thromboxane/prostaglandin (TP) receptor can be observed in a human cultured podocyte cell line, as well as in podocytes from streptozotocin (STZ)-induced diabetic mice. This upregulation contributes to mitochondrial excessive fission and podocyte injury through the activation of the Rho-associated kinase 1 (ROCK1)-DRP1 signaling pathway, promoting the phosphorylation of DRP1 at the Ser637 site (Liu S. et al., 2022). The lncRNA maternally expressed gene 3 (Meg3) contributes to podocyte injury induced by high glucose *via* increasing the levels of DRP1 and promoting its translocation to mitochondria (Deng et al., 2020). OMA1, a mitochondrial inner membrane zinc metalloprotease, is involved in the proteolysis of OPA1 when mitochondria are stressed, (Ehses et al., 2009), and glomerular OMA1 Zinc Metallopeptidase (OMA1) was activated in a time-dependent manner in DKD (Q et al., 2022). In addition, OMA1 activation-mediated hydrolysis of OPA1 participates in the mitochondrial fusion decrease of HG-induced podocyte (Q et al., 2022). *In vitro* experiments on HK2 cells, phosphoglycerate mutase family member 5 (PGAM5) exacerbates diabetic renal tubular injury, while AMPK activators mitigate DKD by rescuing the dephosphorylation of DRP1S637 and inhibiting the mitochondria-translocation of DRP1, that is to reduce mitochondrial fission through the AMPK/SP1/PGAM5 pathway (Liu et al., 2020).

### Mitochondrial mitophagy dysfunction

#### What is mitophagy?

Autophagy of damaged mitochondria, called mitophagy, is a crucial organelle quality control process that plays a significant role in the pathogenesis of inflammation, cancer, aging, and age-related diseases (Alula et al., 2023). The term “mitophagy” was first introduced in 2005, (Lemasters, 2005), and within a few years, major breakthroughs made the discovery of critical proteins that selectively mediate mitochondrial degradation (Schweers et al., 2007; Narendra et al., 2008; Sandoval et al., 2008). In mammals, mitophagy can occur through receptor-mediated or Ubiquitin-mediated manner (Onishi et al., 2021). The receptors encompass p62/sequestosome1 (SQSTM1), (Geisler et al., 2010), BCL2 interacting protein 3 (BNIP3), (Hanna et al., 2012), NIP3 like protein X (Nix/BNIP3L), (Novak et al., 2009), FUN14 domain containing 1 (FUNDC1), (Qi et al., 2012), B-cell lymphoma 2-like 13(BCL2L13), (Murakawa et al., 2015; 2019), FK506-binding protein 8 (FKBP8) (Bhujabal et al., 2017) and histone deacetylase 6 (HDAC6) (Lee et al., 2010). These receptor proteins have a special function that interacts with either mammalian Atg8 family members (LC3A/B/C, GABARAP, GABARAP-L1/2) *via* an microtubule-associated protein 1 light chain 3 (LC3) interacting region (LIR) or ubiquitin *via* an ubiquitin-binding domain (UBD) during mitophagy.

#### The ubiquitin-mediated manner in mitophagy: Parkin dependent or independent pathway

**FIGURE 5:**
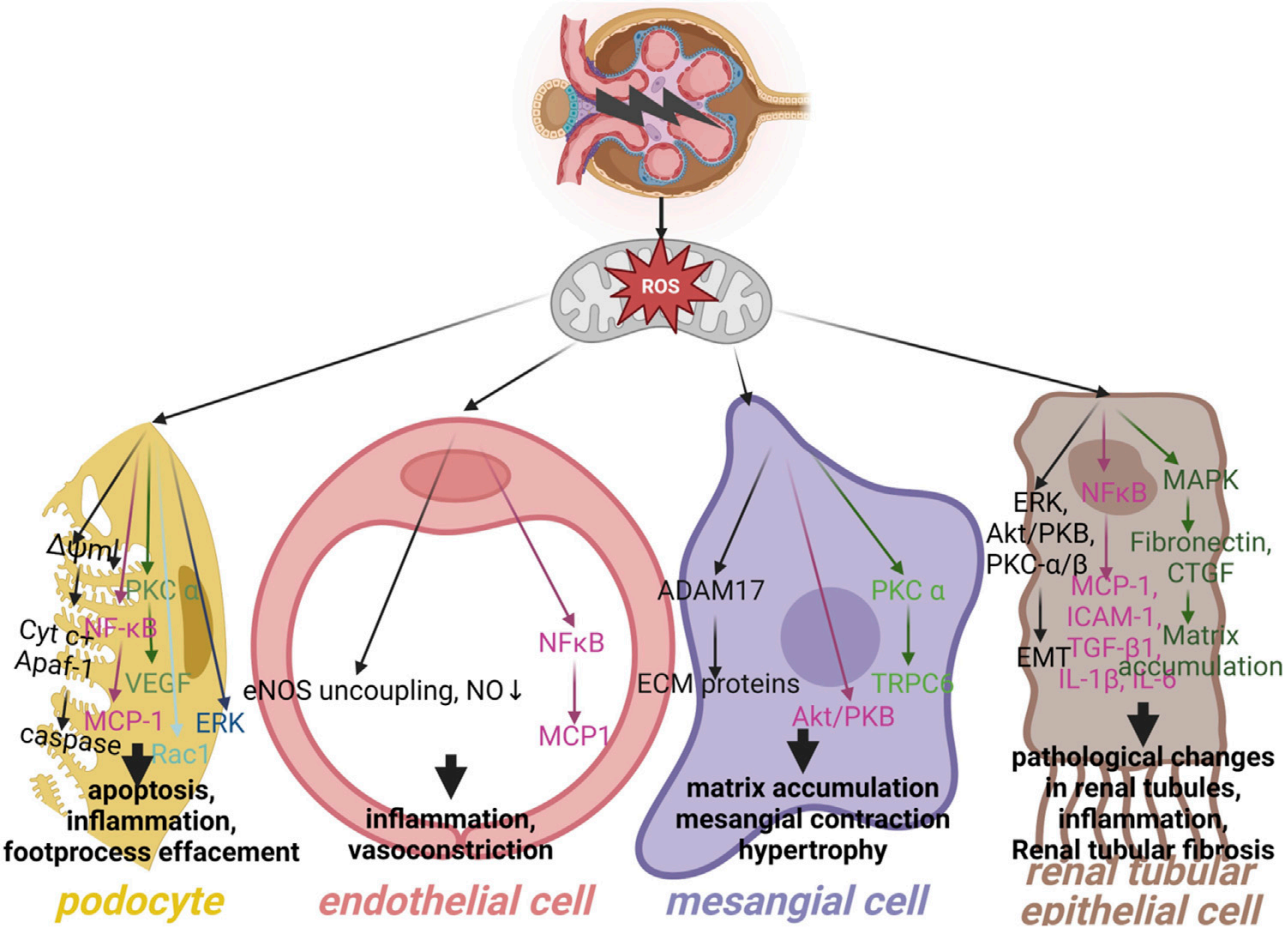
Mitophagy pathways and several pathways regulating mitophagy in a hyperglycemic background. PINK1, PTEN-induced putative kinase 1; OMM, outer mitochondrial membrane; p62/SQSTM1, sequestosome 1; NBR1, NBR1 autophagy cargo receptor; NDP52, calcium binding and coiled-coil domain 2; TAX1BP1, Tax1 binding protein 1; OPTN, Optineurin; LC3, microtubule-associated protein 1 light chain 3; FKBP8, FK506-binding protein 8; FUNDC1, FUN14 domain containing 1; BNIP3, BCL2 interacting protein 3; Nix/BNIP3L, NIP3 like protein; PGAM5, phosphoglycerate mutase family member 5; ULK1, unc-51-like autophagy activating kinase 1; mTOR, mammalian target of rapamycin; AMPK, adenosine monophosphate-activated protein kinase; MFF, mitochondrial fission factor. Created with BioRender.com.

The PTEN-induced putative kinase 1 (PINK1)/Parkin-mediated pathway is the most studied mechanism of mitophagy (Figure 5) (Xiao et al., 2022). Dissipation of mitochondrial membrane potential (ΔΨm) is a potent trigger of mitophagy (Elmore et al., 2001). When the ΔΨm loss, PINK1 accumulates on the outer mitochondrial membrane (OMM) and phosphorylates pre-existing ubiquitin molecules at Ser65, recruiting Parkin (Okatsu et al., 2015; Wauer et al., 2015). PINK1 also phosphorylates the ubiquitin-like domain of Parkin, releasing its catalytic RING2 domain, which stabilizes Parkin in an active state. This leads to the ubiquitination of various OMM proteins, such as voltage-dependent anion channel (VDAC), mitochondrial rho GTPase (MIRO), MFN1, and MFN2 (Gladkova et al., 2018; Mitrofanova et al., 2022). These ubiquitinated proteins recruit mitophagy receptors including p62/SQSTM1, NBR1 autophagy cargo receptor (NBR1), calcium binding and coiled-coil domain 2 (NDP52), Tax1 binding protein 1 (TAX1BP1), and Optineurin (OPTN) and provide signals for Parkin-mediated mitochondrial degradation (Geisler et al., 2010; Jee and Cheong, 2023). In Parkin-independent pathway, PINK1-generated phospho-ubiquitin serves as the autophagy signal on mitochondria and PINK1 can directly recruit autophagy receptors OPTN and NDP52 to promote autophagy (Lazarou et al., 2015). This pathway can be used to compensate for the dysfunction of Parkin-mediated one.

#### The receptor-mediated manner in mitophagy

In this section, we mainly discuss the receptor-mediated manner involving FUNDC1, BNIP3 and Nix (Figure 5). FUNDC1 contains a region interacting with LC3. In conditions such as hypoxia or loss of ΔΨm, FUNDC1 undergoes dephosphorylation at Tyr18 and Ser13 by PGAM5, while Ser17 is phosphorylated by unc-51-like autophagy activating kinase 1 (ULK1). This enhances the interaction between FUNDC1 and LC3, promoting mitophagy. PGAM5 activity is regulated by BCL2-like 1 (BCL-XL) (Wu et al., 2014). FUNDC1 interacts with two crucial regulators of mitochondrial dynamics: DRP1 and OPA1. This interaction allows FUNDC1 to coordinate both mitochondrial dynamics and mitophagy processes (Chen et al., 2016). BNIP3 and Nix, located on the outer mitochondrial membrane, are implicated in stress detection and hypoxia-triggered mitophagy (Zhang and Ney, 2009). Accumulation of ROS (induced by ROS) promotes Nix-mediated mitophagy *via* a recruitment of LC3 to mitochondria (Melser et al., 2013). Both BNIP3 and Nix interact with LC3 to enhance autophagosomal recruitment to mitochondria (Rogov et al., 2017).

#### Impaired mitophagy - A hallmark in DKD

Studies show that as kidney damage progresses in diabetes, ΔΨm decreases in the proximal tubule, (Coughlan et al., 2009; Tan et al., 2010; Forbes et al., 2013), endothelial cells (Qi H. et al., 2017) and podocytes (Qi W. et al., 2017). Podocytes have naturally high mitophagy levels due to maturity, while tubular cells show low baseline levels, increasing during stress (Ito et al., 2022). Inhibition of PINK1 distinctly increases albumin permeability and impairs the mitophagy function of podocytes (Audzeyenka et al., 2022). Impaired mitophagy is recognized as a hallmark of human DKD and of rodent models of DKD (Mitrofanova et al., 2022). In DKD patients and rats with STZ-induced DKD, high glucose levels activate thioredoxin-interacting protein (TXNIP) causing autophagosome accumulation and reduced autophagic clearance in tubular cells (Huang et al., 2016). High glucose-treated renal tubular epithelial cells and biopsies from DKD patients exhibit decreased mitophagy levels (Chen K. et al., 2018). Studies propose that glomerular endothelial cells exhibit decreased levels of LC3-II, PINK1, and Parkin when exposed to high glucose conditions, similar to findings observed in different types of kidney cells (Sun et al., 2019). In a rat model of STZ diabetes, in 4-week after diabetes induction (early diabetes), PINK1 was increased in the renal cortex (Smith et al., 2012). Diabetic db/db mice in 12-week period showed reduced expression of key mitophagy markers including mitochondrial PINK1, Parkin, LC3-II, Beclin-1, and Atg5, indicating impaired mitophagy (Feng et al., 2018). It is predictable that in early diabetes, the kidney activates mitophagy to satisfy the clearance of damaged mitochondria, but as DN progresses, leading to impairment of mitophagy and accumulation of fragmented mitochondria, even to death.

#### Regulation of mitophagy in hyperglycemic conditions

Several pathways regulating mitophagy in a hyperglycemic background are summarized here (Figure 5). Under physiological conditions when fuels are sufficient, AMPK activity is suppressed and mTOR inhibits ULK1, inhibiting mitophagy (Kim et al., 2011; Groenewoud and Zwartkruis, 2013). During nutrient deprivation, the increase of AMP/ATP ratio can activate AMPK and inhibit mTOR, phosphorylating the serine/threonine-protein kinase ULK1 and inducing mitophagy (Bhargava and Schnellmann, 2017). During oxidative stress, AMPK can be activated and inhibit mTOR, again triggering mitophagy (Groenewoud and Zwartkruis, 2013; Melser et al., 2013). A more direct role for AMPK to stimulate mitophagy has also been suggested. AMPK promotes DRP1 recruitment from cytosol to the OMM *via* mediating phosphorylation of MFF on its Ser155 and Ser172 sites (Ducommun et al., 2015; Toyama et al., 2016). Upon recruitment, DRP1 promotes the fission of mitochondria and subsequently enhances mitochondrial mitophagy (Toyama et al., 2016; Zhang and Lin, 2016). Evidences show that dysregulation of AMPK induced by elevated ROS level decreases mitochondrial function in the diabetic kidney (Dugan et al., 2013).

#### Hypoxia-induced mitophagy mechanisms

Other stimuli, such as hypoxia, cause PGAM5 to dephosphorylate its substrate FUNDC1 (Chen et al., 2014). FUNDC1 sequentially interacts with LC3, promoting mitophagy (Liu et al., 2012; Chen et al., 2014). Alternatively, hypoxia can also induce mitophagy through BNIP3 and Nix *via* hypoxia-inducible factor 1α (HIF1α) involving (Novak et al., 2009; Zhang and Ney, 2009). HIF1α can directly promote the transcription of BNIP3 and Nix by binding to BNIP3 promoter and recruiting other co-activator proteins to Nix. However, external stimuli that trigger mitophagy are unknown. Additional studies are needed to clarify the mechanisms that regulate mitophagy in DN.

## Targeted treatment using pharmacological agents

As previously discussed, mitochondrial dysfunction is implicated in various aspects of DKD pathogenesis. Mitochondrial dysfunction can already manifest in the initial stages of hyperglycemia and is recognized as a key factor in the pathogenesis of DKD (Cleveland and Schnellmann, 2023). Despite the incomplete understanding of the precise role of mitochondrial function in DKD, improving mitochondrial dysfunction may be a very promising strategy for treatment of DKD (Yang et al., 2024). Emerging therapeutic approaches targeting mitochondrial dysfunction are currently being explored for the management of CKD. Data from preclinical studies indicate that these strategies may also hold promise for the treatment and prevention of DKD. We will discuss the specific strategies including targeting the redox state of mitochondria, mitochondrial biogenesis and ATP synthesis activators, fission inhibitors, and mitophagy inducers (Table 1).

**TABLE 1:** Mitochondria-targeted therapeutics.

Compounds	Mechanism	References
Antioxidants		
S1QELs	Target the complex I site	Brand et al. (2016)
OP2113	Target the complex I site	Roubenne et al. (2023)
CoQ10	Antioxidant	Persson et al. (2012), Sourris et al. (2012)
Idebenone	Antioxidant, delivers electrons directly to complex III	Jaber and Polster (2015)
EPI-743	Enhances biosynthesis of glutathione; antioxidant	Martinelli et al. (2012)
RP-103	Enhances cellular delivery of cysteine and/or glutathione synthesis	Verny et al. (2017)
Curcumin	Antioxidant	Abd Allah and Gomaa (2015), Yang et al. (2015), Lu et al. (2017)
SKQ1	Antioxidant	Petrov et al. (2016)
MitoQ	Antioxidant concentrate at matrix in a ΔΨm-dependent manner; ROS scavenger	Chacko et al. (2010), Ward et al. (2017)
MitoTEMPO	Inhibit the NLRP1 inflammasome	Liu et al. (2022a)
MTP-131	Prevents the peroxidation of cardiolipin by Cyt C, increase OXPHOS efficiency, potential antioxidant effects	Szeto et al. (2011), Birk et al. (2013), Q et al. (2022)
KH-176	Mitochondrial-targeted antioxidant, enhances OXPHOS	Beyrath et al. (2018)
Biogenesis activators		
AICAR	SIRT3 activator, AMPK agonist	Lempiäinen J et al. (2012)
Metformin	Activate AMPK and upregulate expression and activation of PGC-1α	Hawley SA et al. (2010), Aatsinki SM et al. (2014), Ota S et al. (2009)
Melatonin	Stimulate AMPK activity and PGC1α expression	Li J et al. (2019)
PXL770	Stimulate AMPK	Dagorn PG et al. (2023)
Formoterol	β2-adrenoceptor agonist, increase PGC-1α synthesis	Arif E et al. (2019)
L-BAIBA	Upregulate PGC-1α and TFAM	Audzeyenka I et al. (2023)
Puerarin	Activate AMPK and upregulate the level of PGC-1α	Chen X.-F. et al. (2018)
Catalpol	Activate the AMPK/PGC1α/TFAM pathway	Xu DQ et al. (2020)
Gomisin N	Increase PGC-1α, TFAM, and NRF1	Jung DY et al. (2017)
EGCG	Increase PGC1α	Oliveira et al. (2016)
Epicatechin	Upregulate the expression of SIRT1, TFAM, and PGC-1α	Ramírez-Sánchez I et al. (2016)
Salidroside	Increase SIRT1 and PGC1α	Xue H et al. (2019)
Glycyrrhic acid	Increase SIRT1 and PGC1α	Hou S et al. (2017)
Fenofibrate/Bezafibrate	Increase mitochondrial biogenesis	Scarpulla (2011)
Thiazolidinediones	PPAR agonist	Bogacka I et al. (2005)
Fission inhibitors		
Mdivi-1	Inhibit DRP1 and induce mitochondria fusion	Cassidy-Stone A et al. (2008)
P110	Inhibited GTPase activity of DRP1 and its interaction with FIS1	Qi X et al. (2013)
DRP1i27	Inhibited GTPase activity of DRP1	Rosdah AA et al. (2022)
Polydatin	Decreasing phosphorylation of DRP1	Ni Z et al. (2017)
D-glucaric acid	Attenuate mitochondrial fission	Zhan M et al. (2015)
Exenatide	Inhibited the mitochondrial localization of DRP1	Torres G et al. (2016)
Berberine	Inhibiting DRP1 activation	Qin X et al. (2019)
Dynasore	Inhibited GTPase activity of Dynamin 1, Dynamin 2, DRP1	Macia E et al. (2006)
1H-pyrrole-2-carboxamide compounds	Inhibited GTPase activity of DRP1	Lim JW et al. (2020)
Fusion stimulators		
Enzyme (HO-1)	Upregulated MFN1/2 expression	Hull TD et al. (2016)
Melatonin	Activated the Notch1/MFN2 signaling pathway, upregulated MFN2 expression	Pei H et al. (2016)
M1	Stimulated Mitofusins	Wang D et al. (2012)
15-oxospiramilactone (S3)	Deubiquitinated MFN1/2, augmented the activity of MFN1/2	Yue W et al. (2014)
Punicalagin	Stimulated OPA1	Fu F et al. (2021)
κ-opioid receptor	Stimulated OPA1	Wang K et al. (2020)
Paeonol	Stimulated OPA1	Liu C et al. (2021)
Mitophagy inducers		
Melatonin	Upregulation of NF-κB (preceded by Akt) and Nrf2 to induce mitophagy	Sun B et al. (2018)
Pioglitazone	NF-κB to induce mitophagy	Yoon YM et al. (2018)
Rapamycin	mTOR inhibition to induce Parkin-mediated mitophagy	Wang Y. et al. (2018)
Metformin	mTOR inhibition and SIRT3 upregulation to induce Parkin-mediated mitophagy	(Wang C. et al., 2018; Chen L et al., 2020)
Spermidine	mTOR axis modulation to induce Parkin mitophagy	Qi Y et al. (2016)
Salidroside	Modulate mTOR axis *via* ATM-dependent Parkin/PINK1 pathways	Li R et al. (2019)
Honokiol	Trigger SIRT3 (*via* the AMPK-PGC-1α) pathway to induce mitophagy	Wang J et al. (2018)
Astragaloside II	Upregulate Nrf2 and PINK1	Su J et al. (2021)
Src inhibitor PP2	Inhibit FUNDC1 phosphorylation	Zheng T et al. (2022)
MitoQ	Reverse deficient mitophagy	Xiao L et al. (2017)

### Targeting the redox state of mitochondria

#### The clinical efficacy of quinone antioxidants in mitochondrial diseases: limited effect despite preclinical rationale

Oxidative stress is an important component in the development of DKD. Quinone-based antioxidants, encompassing CoQ analogs idebenone and EPI-743, (vatiquinone, vincerinone) alongside KH176, are employed as a frontline pharmacological agents against mitochondrial oxidative stress. Coenzyme Q10 (CoQ10, ubiquinone) is a component of the mitochondrial respiratory chain with antioxidant propertiy. CoQ10 is renoprotective and prevents damaging changes in mitochondrial function and morphology in rodent models of DKD (Sourris et al., 2012). CoQ10 has reached phase III clinical trials for mitochondrial disorders (University of Florida, 2017), but substantial benefits of the therapy have not been reported to date. Idebenone influences mitochondrial function acting as an antioxidant and directly transfers electrons to complex III bypassing complex I of the ETC (Jaber and Polster, 2015) This agent is already approved in Europe for indications such as Leber hereditary optic neuropathy, (Klopstock et al., 2013), but phase II and phase III clinical studies have not reached primary end points in other mitochondrial diseases (Lagedrost et al., 2011). EPI-743 enhancing glutathione biosynthesis has showed beneficial effects on mitochondrial diseases (Martinelli et al., 2012). In contrast, RP-103 (cysteamine bitartrate) has the function of enhancing the cellular supply of cysteine, which is thought to facilitate glutathione synthesis and has entered into phase II/III clinical trials in children with inherited mitochondrial diseases (Verny et al., 2017). KH-176, acting as a ROS-Redox modulator, is also thought to have antioxidant properties and can effectively increase the maximal efficiency of OXPHOS complexes I and IV by targeting the Thioredoxin/Peroxiredoxin system (Beyrath et al., 2018). This compound has completed pharmacokinetic studies and a phase I clinical trial (Koene et al., 2017). In the KHENERGY clinical trial, KH-176 shows no improvement in mitochondrial diseases (Khondrion, 2018).

#### Mitochondria-targeted antioxidants: a promising yet unproven therapeutic strategy for diabetic complications

Despite the promising potential of these agents, the efficacy of these antioxidants is constrained and clinical studies in patients with diabetic complications have been mostly disappointing (Forbes et al., 2008; Sorrentino et al., 2018). A probable explanation is that transport of these agents into the mitochondria, which is a highly lipophilic environment, is not sufficient to achieve efficacy or that the wrong mitochondrial is targeted. The advent of mitochondria-targeted antioxidants (e.g. Mitoquinone (MitoQ), (Graham et al., 2009), MitoTEMPO, MitoE, Mito-CP, SkQ1 (Petrov et al., 2016) and SkQR1) are attempts to reduce mitochondrial oxidative stress. Administration of MitoQ prevented DKD (Chacko et al., 2010) in a murine model of inherited diabetes and in experimental type 2 DM (T2DM) model (Ward et al., 2017). MitoQ was shown to be safe in clinical trial with Parkinson’s disease, fatty acid disease. The effect of MitoQ under clinical trial (NCT02364648) for CKD show no results posted. MitoTEMPO, improves podocyte injury by inhibiting the NLRP1 inflammasome and promoting the PINK3/Parkin pathway-mediated mitophagy (Liu B. et al., 2022). Szeto-Schiller (SS) peptide family (also known as MTP-131, SS-31, elamipretide or Bendavia) also could directly target cardiolipin peroxidation, independent to mitochondria membrane potential (Birk et al., 2013). MTP-131 passively diffusing into the mitochondria and residing in the IMM, has shown beneficial effects on function and fibrosis in acute kidney injury (AKI) studies involving ischaemic–reperfusion injury, models of experimental DN and unilateral ureteral obstruction (Q et al., 2022; Szeto et al., 2011; Birk et al., 2013). Further studies are needed to determine the effect of MitoQ and MTP-131 in human diabetic kidney disease. S1QELs and OP2113, inhibit ROS production without disturbing normal OXPHOS by targeting the complex I site (Brand et al., 2016; Roubenne et al., 2023). Curcumin (a derivative of turmeric) has an antioxidant effect *via* various pathways relevant to the development of DKD (Yang et al., 2015; Lu et al., 2017). In rat models of DKD, curcumin treatment shows modest improvements in renal function (Abd Allah and Gomaa, 2015). However, a pilot study showed no effects of curcumin treatment for 8 weeks on proteinuria and estimated GFR in patients with DKD (Jiménez-Osorio et al., 2016). Although mitochondria-targeted antioxidants may help reduce oxidative damage and repair lipid membranes, they may not restore functionality to damaged mitochondria.

#### Other experimental approaches have also been tested

Mitochonic acid 5 (MA-5), deriving from the plant growth hormone indole3-acetic acid, can protect mitochondrial function by regulating mitochondrial ATP synthesis and reducing the level of mitochondrial ROS without affecting activity of mitochondrial complexes I–IV (Suzuki et al., 2016; Lei et al., 2018). Astragaloside IV ameliorates mitochondrial dysfunction by upregulated Nrf2-ARE/TFAM signaling, then decreasing ROS production in mouse podocytes line (Shen et al., 2023). These agents, however, remain in their early stage of exploration.

### Induction of mitochondrial biogenesis

Several pharmacological agents have been identified to have effects on these regulatory points, thereby enhancing mitochondrial biogenesis. Agents that promote mitochondrial biogenesis are an potential therapeutic strategy for DKD (Komen and Thorburn, 2014).

#### Targeting upstream sensor - AMPK

AMPK acts as an energy sensor of the cell and works as a key regulator of mitochondrial biogenesis. Pretreatment with AICAR, an AMPK activator, attenuated injury and tubular necrosis and improved renal function in a rat I/R induced AKI (Lempiäinen et al., 2012). Metformin, widely known for its role in diabetes management, specifically blocks the functioning of mitochondrial ETC complex I, bolsters mitochondrial biogenesis by subsequently activating AMPK, leading to an upsurge in PGC-1α expression and activation (Ota et al., 2009; Hawley et al., 2010; Aatsinki et al., 2014). Another study showed that melatonin (N-acetyl-5-methoxytryptamine) could also stimulate AMPK activity and PGC-1α expression to protect mice from diabetic kidney injury (Li et al., 2019). PXL770 is a clinical stage direct and specific allosteric AMPK activator and instigates mitochondrial biogenesis by acting as a direct stimulant of AMPK. In a preclinical investigation, PXL770 treatment restores mitochondrial DNA copy number and PGC-1α mRNA expression in mice with autosomal dominant polycystic kidney disease (ADPKD) (Dagorn et al., 2023). Nicotinamide riboside reveal SIRT2’s protective role in a diabetic-dietary rat model of NASH *via* deacetylation of NLRP3, LKB1, and FOXO3a and restoration of AMPK-ACC/PGC-1α-Nrf2 signaling (Hamad et al., 2025). Treatment of db/db mice with type 2 diabetes with nicotinamide riboside prevented several manifestations of kidney dysfunction (i.e., albuminuria, increased urinary kidney injury marker-1 (KIM1) excretion, and pathologic changes) (Myakala et al., 2023).

#### Targeting upstream sensor - SIRT1

PGC-1α is a marker of mitochondrial biogenesis and SIRT1 is known as an upstream activator of PGC-1α. Studies show downregulation of SIRT1 in podocytes and glomeruli in human diabetic kidneys as well as in diabetic mice (Chuang et al., 2007; Liu et al., 2014). Furthermore, selective pharmacological activation of SIRT1 in cultured podocytes increased SIRT1-mediated PGC-1α activity and protected cells from mitochondrial dysfunction resulting from hyperglycemic condition (Hong et al., 2018). Nihalani et al. showed that the β2-adrenergic receptor agonist Formoterol increases the expression of PGC-1α and multiple ETC proteins, promoting podocyte recovery after glomerular injury (Arif et al., 2019). Besides, β-Aminoisobutyric acid (L-BAIBA) upregulates PGC-1α and TFAM, promoting mitochondrial biogenesis and ameliorating podocyte injury (Audzeyenka et al., 2023).

#### Targeting downstream effecter - PPARγ

Studies has also shown that agonists of PPARγ stimulate mitochondrial biogenesis (Yang et al., 2009; Corona and Duchen, 2016). Bezafibrate and fenofibrate have been shown to target PPARα, which collaborates with PGC-1α to enhance mitochondrial biogenesis (Scarpulla, 2011). Thiazolidinediones, promote mitochondrial biogenesis by binding to and activating PPARs, which heterodimerize with Retinoid X Receptor (RXR) and bind to PPAR response elements in the DNA, thereby upregulating the transcription of genes involved in mitochondrial biogenesis (Bogacka et al., 2005).

#### Some natural products enhancing mitochondrial biogenesis

Multiple studies have explored natural products for their role to increase mitochondrial biogenesis. Puerarin, an isoflavonoid compound primarily from the roots of Pueraria lobata, significantly activates AMPK and upregulates the level of PGC-1α, thereby stimulating mitochondrial biogenesis (Chen X.-F. et al., 2018). Catalpol, an iridoid glycoside component, promotes mitochondrial biogenesis through activating the AMPK/PGC-1α/TFAM pathway (Xu et al., 2020). Gomisin N, a natural lignan compound primarily isolated from Schisandra chinensis, can upregulate genes linked to mitochondrial biogenesis, such as PGC-1α, TFAM, and NRF1, *via* phosphorylating AMPK and Protein kinase B (Akt) (Jung et al., 2017). Green tea extracts also increase PGC-1α and protect mice against cyclosporine-induced renal injury (Rehman et al., 2014). Catechins and their gallate esters constitute a group of polyphenolic compounds which include epicatechin, epicatechin gallate, epigallocatechin and epigallocatechin gallate (EGCG) (Farhan, 2022). EGCG increases the expression of PGC-1α at both mRNA and protein levels, leading to an increased mitochondrial biogenesis (Oliveira et al., 2016). Epicatechin, upregulates the expression of SIRT1, TFAM, and PGC-1α, resulting in increased mitochondrial biogenesis in mice and human coronary artery endothelial cells (Ramírez-Sánchez et al., 2016). Some other agents have also shown impacting PGC-1α activity *via* less clear mechanisms. For example, Salidroside, the active component of the Rhodiola rosea plant, and glycyrrhic acid from liquorice root both increase SIRT1 and PGC-1α and protect mice from DN (Xue et al., 2019; Hou et al., 2017). Many of the above species lack the necessary specificity for modulating PGC-1α. Treatment with sacubitril/valsartan (Sac/Val) improved mitochondrial function in maintaining mitochondrial biogenesis and fatty acid oxidation in db/db kidneys (Myakala et al., 2021). These compounds, through their action on specific targets, highlight the potential for pharmacological intervention to promote mitochondrial biogenesis in the context of mitochondrial dysfunction. Nevertheless, their clinical translation still requires further investigation.

### Targeting mitochondrial dynamics

The imbalanced mitochondrial dynamics are associated with various diseases which are extensively characterized with deficiencies in mitochondrial function. It is demonstrated that improving mitochondrial health by modulating mitochondrial dynamics can reduce the risk of disease and promote overall wellbeing. Proteins involved in mitochondrial dynamics were altered in diabetic mice, favoring a pro-fission mitochondrial state in diabetic background (Cleveland et al., 2020). In recent years, research has made significant progresses in the discovery and development of effective inhibitors targeting mitochondrial fission.

#### Regulating mitochondrial fission

Mitochondrial division inhibitor specifically targets mitochondrial division proteins, selectively inhibiting the function of DRPs by interacting primarily with an orthosteric domain (Cassidy-Stone et al., 2008). Mdivi-1, as the initial inhibitor that specifically targets mitochondrial division proteins, can restore mitochondrial morphology in disease models characterized by excessive mitochondrial fission. As a small peptide, P110 specifically inhibits the interaction between FIS1 and DRP1, and suppresses mitochondrial fission process (Qi et al., 2013). DRP1i27, an inhibitor of human DRP1, binds to the GTPase domain of DRP1 by forming hydrogen bonds with Asp218 and Gln34 (Rosdah et al., 2022). Polydatin is resveratrol glycoside and is extracted from the radix of Polygonum cuspidatum. In conditionally immortalized mouse podocytes under high glucose condition, Polydatin attenuated mitochondrial fission by decreasing phosphorylation of DRP1 at Serine-616 (Ni et al., 2017). Administration of D-glucaric acid, which reduced ROS by inhibiting MIOX, to human (HK-2) and porcine (LLC-PK1) renal proximal tubular cells under diabetic background proved to attenuate renal mitochondrial fission, oxidative stress, while promoting the restoration of renal mitochondrial fusion (Zhan et al., 2015). Exenatide suppresses mitochondrial fission by phosphorylating DRP1 at Ser-637, thereby preventing the localization of DRP1within the mitochondria (Torres et al., 2016). Pretreatment of berberine in diabetic db/db mice significantly reverses elevated glucose, podocyte damage, and mesangial matrix expansion by inhibiting DRP1 activation (Qin et al., 2019). MitoQ protects podocytes against dysfunction and injury, such as Ang II-induced mitochondrial fission, by modulating the Keap1- NRF2 signaling pathway (Zhu et al., 2021). Several other small molecules, such as dynasore (Macia et al., 2006) and 1H-pyrrole-2-carboxamide compounds (Lim et al., 2020), have been reported to inhibit DRP1 and reduce mitochondrial fission. These newly identified molecules have demonstrated potential in preclinical research, but additional study is needed to evaluate their safety and efficacy in humans.

#### Regulating mitochondrial fusion

Chemical therapeutics have demonstrated potential in restoring abnormal mitochondrial dynamics by promoting and regulating the mitochondrial fusion machinery. However, there is a lack of reported small molecule compounds that directly affect mitochondrial fusion proteins. These small molecule compounds promote mitochondrial fusion *via* OPA1-mediated upregulating MFN1/2 expression or stimulating OPA1 mechanism. These compounds include enzyme (HO-1), (Hull et al., 2016), Melatonin, (Pei et al., 2016), M1, (Wang et al., 2012), 15-oxospiramilactone (S3) (Yue et al., 2014) in the former category and Punicalagin, (Fu et al., 2021), κ-opioid receptor, (Wang et al., 2020), Paeonol (Liu et al., 2021) in the latter category.

### Targeting mitophagy

Numerous drugs have been reported that they can improve mitophagy dysfunction. Melatonin has been demonstrated to increase phosphorylation of Akt and NF-κ B, which subsequently activates PINK1-dependent protective mitophagy. Additionally, melatonin may upregulate NRF2-induced mitophagy to protect neuron in subarachnoid haemorrhage (SAH) against apoptosis (Sun et al., 2018). Pioglitazone, an anti-diabetic drug for T2DM in the category of thiazolidinediones (TZDs), boots PINK1 expression *via* NF-κ B activation and promotes mitophagy (Yoon et al., 2018). Rapamycin, a compound approved by the FDA, promotes Parkin/PINK1 mitophagy, probably *via* p62, to restore mitochondrial homeostasis (Wang et al., 2018). Metformin, an mTOR inhibitor, promotes mitophagy through various signaling pathways, including the AMPK- NRF2 as well as SIRT3 pathway (Wang C, et al., 2018; Chen L. et al., 2020). Recent randomized controlled clinical trials have demonstrated its potential to enhance mitophagy in T2DM (Wang C. et al., 2018). Spermidine induces mitophagy *via* ATM-dependent Parkin/PINK1 pathway, which modulate of the mTOR axis (Qi et al., 2016). Salidroside, a plant extract, has been demonstrated to protect dopamine (DA) neurons in Parkinson’s disease (PD) models by enhancing mitophagy. The underlying mechanism is likely linked to its bioactive effects on DJ-1/NRF2 pathway (Li and Chen, 2019). Honokiol, an agonist of SIRT3, promotes mitophagy and mitochondrial dynamics *in vitro* in an SIRT3-dependent manner *via* the AMPK/PGC-1α signaling pathway, and its neuroprotection has been validated *in vivo* (Wang J. et al., 2018). Gui et al. reported that Astragaloside II ameliorates podocyte injury and mitophagy dysfunction in diabetic rats by enhancing the expression NRF2 and PINK1 (Su et al., 2021). Sun et al. discovered that the Src inhibitor PP2 inhibits the phosphorylation of FUNDC1and promotes mitophagy, thereby protecting podocytes from mitochondrial damage (Zheng et al., 2022). Additionally, MitoQ alleviates tubular injury in DKD *via* NRF2/PINK1 (Xiao et al., 2017). Moreover, because mitophagy is dependent on mitochondrial dynamics, it is reasonable that modulation of fission and fusion may target the process of mitophagy. Preclinical studies are required to investigate efficacy and possible adverse effects of these chemicals in DN.

However, despite the many available and promising options for recovering mitochondrial dysfunction in DKD or hyperglycemic background, a deeper understanding of the therapeutic mechanisms and more further researches of these components are necessary to achieve precise treatment of DKD.

## Future research directions

On the therapeutic aspect, although we have demonstrated that improving mitochondrial function can delay the progression of DKD in some cellular or animal experiments, a deeper evaluation of its therapeutic efficacy and safety is still lacking, so there are no drugs targeting mitochondrial dysfunction in DKD yet. The journey from bench to bedside is full of challenges, including the clinical heterogeneity of mitochondrial disorders, the low clinical efficacy and the potential side effects of long-term pharmacological interventions. Concurrently, the necessity for the conception of highly precise therapies becomes increasingly evident and urgent. Future studies should prioritize: (1) conducting large-scale clinical trials to evaluate mitochondrial-targeted therapies, focusing on patient-centered outcomes; (2) mitochondrial transplantation is another strategy burgeoning on therapeutic innovation. While most studies reported favorable results after mitochondrial transplantation, validation of the underlying mechanisms pertinent to its clinical translation remains an open challenge; (Hayashida et al., 2021); (3) developing biomarkers for mitochondrial dysfunction to guide personalized treatment; and (4) exploring combination therapies (e.g., SGLT2 inhibitors with mitophagy inducers) to enhance efficacy while minimizing off-target effects. Addressing these gaps will accelerate the translation of mitochondrial therapies into clinical practice.

## Conclusion and perspective

In conclusion, mitochondrial dysfunction constitutes a cornerstone in the pathogenesis of DKD, with multifaceted mechanisms including impaired biogenesis, dysregulated mitophagy, altered fission-fusion dynamics, increased inflammation, altered OXPHOS, and mitochondrial DNA (mtDNA) damage. Understanding these intricate pathways provides crucial insights into potential therapeutic avenues.

Addressing mitochondrial dysfunction in DKD involves a comprehensive approach targeting various aspects of mitochondrial biology. Lifestyle modifications, encompassing regular exercise and dietary adjustments, present promising avenues to alleviate mitochondrial dysfunction and curb DKD progression. Moreover, timely interventions directed at restoring mitochondrial homeostasis through pharmacotherapy, focusing on mitigating oxidative stress and preserving mitochondrial dynamics, offer additional treatment modalities.

However, the complexity of mitochondrial involvement in DKD warrants further investigation to delineate precise mechanisms and develop targeted therapeutic strategies. Future research endeavors should explore innovative approaches aimed at preserving mitochondrial function and alleviating DKD burden, ultimately advancing the management and prognosis of this debilitating condition.
